# Supplementary material for: Factors Related to the Progression of Clinically Isolated Syndrome to Multiple Sclerosis: A Retrospective Study in Lithuania
Source: Medicina (Kaunas). 2022 Aug 30;58(9):1178. doi: 10.3390/medicina58091178 (PMC9500688; doi:10.3390/medicina58091178)
Supplement: Supplementary file 1 [file medicina-58-01178-s001.zip › medicina-1842407-supplementary.pdf]

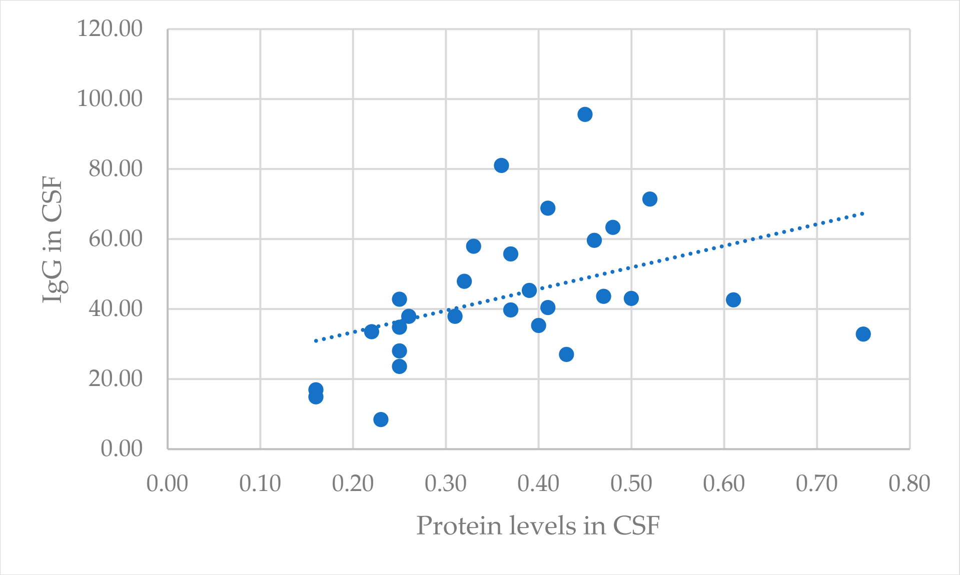

Figure S1. Correlation between IgG levels in CSF and protein levels in CSF.

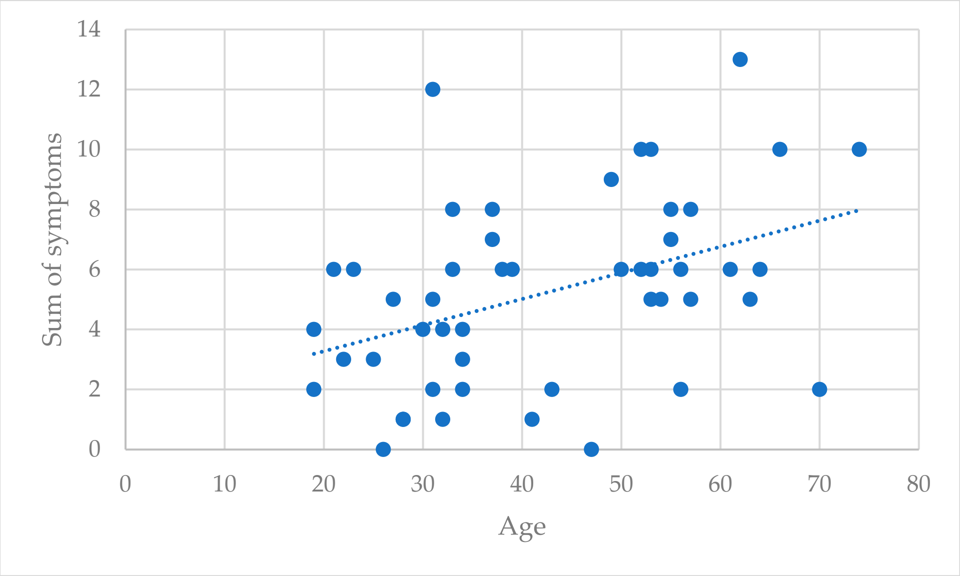

Figure S2. Correlation between patients' age and their symptoms

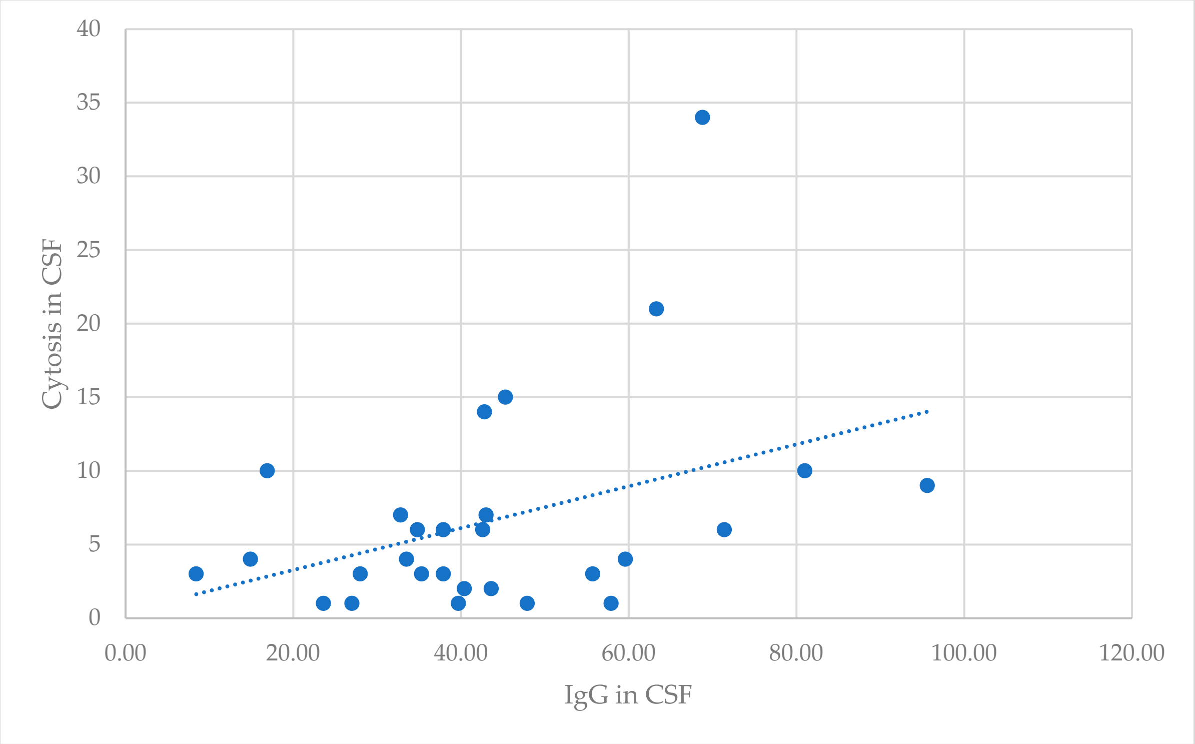

Figure S3. Correlation between IgG levels in CSF and white blood cell count in CSF
